# Continuous local infiltration analgesia is equal to femoral and sciatic nerve block for total knee arthroplasty

**DOI:** 10.1007/s00402-024-05641-7

**Published:** 2025-01-23

**Authors:** Christoph Simon, Matthias Schwab, Hanns Ackermann, Lukas Krüerke, Dirk Meininger

**Affiliations:** 1 Department of Anaesthesia, Main-Kinzig-Kliniken, Herzbachweg 14, 63571 Gelnhausen, Germany; 2 Department of Orthopedic Surgery, Main-Kinzig-Kliniken, Herzbachweg 14, 63571 Gelnhausen, Germany; 3 Department of Biostatistics, Goethe-Universität, Theodor Stern Kai 7, 60590 Frankfurt am Main, Germany; 4 Department of Anaesthesia, SHG Kliniken, Trierer Straße 148, 66663 Merzig, Germany; 5 Department of Anaesthesia, Goethe-Universität, Theodor Stern Kai 7, 60590 Frankfurt am Main, Germany

**Keywords:** Local infiltration analgesia, Total knee arthroplasty, Femoral nerve block, Sciatic nerve block, Pain control

## Abstract

**Background:**

Total knee arthroplasty (TKA) is associated with moderate to severe postoperative pain. Pain control is crucial for rapid mobilisation and reduces side effects as well as the length of hospital stay. In this context, a variety of multimodal pain control regimes show good pain relief, including several nerve blocks, iPACK and local infiltration analgesia (LIA). To compare the analgesic potency of LIA and the combination of continuous femoral nerve block + sciatic single-shot nerve block under general anaesthesia, we conducted a prospective, randomized, controlled, non-blinded single-centre study.

**Method:**

139 ASA I-III Patients were enrolled in the study, randomised into two groups. The LIA group received an intra- and periarticular infiltration containing a mix of ropivacaine 0,2%, adrenaline and ketorolac, followed by an infusion of the same mixture for 48 h via an intraarticular catheter. The patients in the FEM group received a combination of continuous femoral nerve block with a catheter using 30 ml prilocaine 1% and ropivacaine 0,2% plus a single-shot sciatic nerve block via an antero-medial approach (landmark-based technique) with 20 ml ropivacaine 0,75%. Postoperative pain scores were analysed during the first two postoperative days, as well as opioid consumption, the degree of knee movement and the occurrence of infections in both groups applying the Wilcoxon-Mann-Whitney test, Friedman chi-square test and the Log-rank-test.

**Results:**

No significant difference in pain scores, opioid consumption, time to first rescue analgesia, knee range of motion, age, height, weight and ASA could be detected. No severe side effects, such as secondary bleeding or infections, were reported.

**Conclusion:**

Both techniques are well established, provide equal pain relief for TKA and support early postoperative mobilisation.

**Trial Registration:**

DRKS 00027145 08/12/2021. “retrospectively registered”.

## Introduction

In recent years, a growing number of patients have suffered from gonarthrosis. When conservative methods of treatment fail, total knee arthroplasty (TKA) is a common treatment modality for osteoarthritis of the knee joint [1, 2]. Due to major injury caused by the operation, moderate to severe pain during the early postoperative period is to be expected [3–6]. Severe postoperative pain leads to decreased mobility and immobility- and pain-related side effects such as deep vein thrombosis [7, 8], as well as prolonged hospitalization [9, 10]. Insufficient pain control might lead to long-term effects such as chronic pain [11]. This also bears a potential risk of dependence and abuse of opioids. Due to these risk factors, and in the light of the current financial pressure, a sufficient pain control strategy is essential and supports early mobilisation, including physiotherapy, a reduction of side effects and reduced time to discharge from hospital [2, 12, 13].

Common pain control regimes include epidural analgesia (EA), patient-controlled intravenous analgesia with opioids (PCIA) and different techniques of regional anaesthesia [14, 15]. These involve femoral nerve block, sciatic nerve block, saphenous nerve block [16], adductor canal block [17] and iPACK (Interspace between the Popliteal Artery and the Knee) [10, 18] as well as local infiltration analgesia (LIA) in combination with oral analgesics. Each of them is associated with its individual risks and side effects such as nausea, vomiting, sedation, hypotension, motor deficit, infection, urinary retention and nerve injury.

In recent years, the combination of a femoral nerve block + proximal sciatic nerve block has proved to be a viable option for the treatment of postoperative pain and is used widely [12, 13, 19]. The combination of continuous femoral nerve block + single-shot sciatic nerve block provides sufficient pain relief for TKA [20, 21]. The most obvious disadvantage is the motor block, which leads to prolonged immobilisation [20]. It also bears the risk of falls within the first few postoperative days. Nevertheless, we chose the femoral block because it was well established in our institution at that time.

Local infiltration analgesia is performed by the surgeon during the operation. It shows good pain relief without motor weakness and a lower risk of intravascular injection and nerve damage. Since Kerr and Kohan [22] introduced LIA for hip and knee replacement, various studies have been performed to prove the efficacy of LIA [23]. The results of these studies are inconsistent. The question of the most effective strategy remains debatable [24]. However, to our knowledge, no prospective randomized trial comparing the efficacy of LIA with the combination of continuous femoral nerve block plus single-shot sciatic nerve block under general anaesthesia existed in the literature when this trial was initiated [25].

We conducted a randomized, controlled, non-blinded single-centre trial to fill this knowledge gap by investigating potential differences in efficacy between the two methods. We expected the LIA to be as effective as the femoral nerve block, or even more consistent in its effectiveness.

## Methods

Prior to the study, the local ethics review board gave its approval (FF 12/2015), and the study was performed according to the Declaration of Helsinki (Version 2013). From 01.01.2016 to 31.12.2017, we enrolled 139 patients undergoing TKA into the study as per the protocol, while the intention-to-treat population was a minimum of 47 patients per group. After written informed consent was obtained, all patients were randomized into either the Femoralis group or the LIA group when the patients met the inclusion criteria. For inclusion and exclusion criteria see Table 1. For the randomization, we used BIAS for Windows (Version 11, 2015). On the basis of the randomisation list, the patients were allocated to one of the two groups when presenting to the anaesthesia premedication office after giving consent to participate in the trial.

**Table 1:** Inclusion and exclusion criteria

Inclusion criteria	Exclusion criteria
Age > 18 years	Age < 18 years
ASA I–III	ASA > III
Total knee arthroplasty	Trauma
Arthritis of the knee joint	Unicompartimental knee replacement
	Emergency- or second look operations
	Allergy to local anaesthetics
	Preoperative opioid intake
	Missing informed consent

### Procedures and intraoperative management

As the primary outcome parameter, we defined pain scores at rest and in motion. Pain scores (NRS = Numeric Rating Scale) were analysed for the anterior and posterior aspect of the knee separately, as the anterior aspect of the knee is innervated by the femoral nerve, and the posterior aspect of the knee is innervated by the sciatic nerve. Secondary outcomes were defined as total opioid consumption, bleeding, infections, nerve damage and knee range of motion (ROM).

Prior to the operation, all patients received a single dose of ibuprofen 600 mg + oxycodone 10 mg per os, when leaving the ward, no patient received dexamethasone, cox-2-inhibitors nor paracetamol. This was the standard pain management procedure in our institution at this time.

Standard monitoring was applied and recorded at 5-min intervals. An intravenous antibiotic (1500 mg of cefuroxime) was given prior to the operation and an intravenous PCIA [PEGA® PCA, Venner, Germany] with piritramide (1 ml = 1 mg) was connected to a second intravenous catheter. This was done to provide basic analgesia and as per the requirements of the local ethics review board. General anaesthesia was induced with propofol 1%, fentanyl and a muscle relaxant and maintained with sevoflurane. No patient received a spinal anaesthesia.

### Femoralis group

The patients of the Femoralis group received a continuous femoral nerve block + single-shot sciatic nerve block prior to general anaesthesia. The femoral nerve catheter [PLEXOLONG NANO LINE®, 19G × 50 mm, PAJUNK, Germany] was administered by using ultrasound [M-TURBO®, Sono Site, Germany] and nerve stimulation for dual guidance [INNERVATOR 252, Fisher & Paykel and Stimuplex® HNS 12, B. BRAUN, Melsungen, Germany] after injection of a single dose of 30 ml prilocain 1%. Then, the catheter was connected to a PCA pump, filled with 200 ml of ropivacaine 0.2% (5 ml/10 mg per hour, 5 ml/10 mg bolus, lock-out-time 30 min). The sciatic single-shot nerve block was performed by using nerve stimulation only via a ventral approach as a landmark-based technique. A single dose of 20 ml ropivacaine 0.75% was injected via a [UniPlex NanoLine® 22G × 120 mm, PAJUNK, Germany] needle. Both blocks were carried out by either the head of the department of anaesthesiology or his deputy director. The effectiveness of the blocks was verified by testing for sensation of coldness.

### Local infiltration analgesia group

In contrast to the Femoralis group, the patients in this group received a LIA with a mixture of 150 ml ropivacaine 0.2% + 1 mg adrenaline + 30 mg ketorolac, similar to the mixture used by Kerr & Kohen [22], administered in three steps by one of the two surgeons during the operation. Adrenaline was added to the LIA mixture to prolong local anaesthetic uptake and to reduce plasma levels [26]. Furthermore ketorolac was added to reduce opioid consumption [27].

The surgeons injected 50 ml of the solution into the posterior aspect of the knee joint, 50 ml into the medial and lateral aspect and 50 ml subcutaneously, leading to an intraarticular and periarticular injection. This procedure was followed by an intraarticular catheter [Silver Soaker® Infusion Catheter 19G × 61 /6.5 cm, B. Braun, Melsungen, Germany], connected to an elastomeric pump [Pain Buster® 270 ml infusion pump, B. Braun, Melsungen, Germany], filled with a mixture of 250 ml ropivacaine 0.2% + 1 mg adrenaline + 30 mg ketorolac. The infusion rate was limited to 5 ml per hour by the catheter itself; therefore, the elastomeric pump was running for about 50 h. Both groups received 1 g of tranexamic acid prior to the operation and a second dose 3 h after the first one. At the end of the operation, none of the patients had a compression bandage nor a cold pack.

### Postoperative management

Pain scores (NRS, Numeric Rating Scale) at the time of arrival in the recovery room at rest and every full hour were obtained separately for the ventral and posterior aspect of the knee until the patient was discharged. No patient was discharged with a pain score over 3/10. Blood loss, opioid consumption (piritramide in milligrams) and the time to discharge from the recovery room were recorded. On postoperative days (POD) 1 and 2, pain scores, opioid consumption (piritramide in milligrams), consumption of local anaesthetic and blood loss were recorded on the surgical ward by 08:00 and 20:00. In addition, the physiotherapist recorded the pain score at rest before, during and after physiotherapy, as well as the ROM. This was also performed on the surgical ward by the physiotherapist during the day, mostly late in the morning.

For our study, we decided to follow the established local pain treatment concept in our institution. All patients received 10 mg oxycodone 1–0–1 as well as 600 mg of ibuprofen 1–1-1 on the ward. No paracetamol was given.

All patients used the PCIA for rescue medication. The pump contained piritramide 1 mg per ml. All patients were allowed to self-medicate with 1 mg every 6 min with a limit of 10 mg per hour and 15 mg in 4 h. On POD 2, the wound drainage and the LIA catheter were removed by the surgeons. From this moment on, the patients of the LIA group were treated with ibuprofen, oxycodone and the PCIA only. The patients of the Femoralis group were additionally treated with the femoral catheter until the pain intensity was ≤ 3/10 on the numeric rating scale. Differences in pain intensity and opioid consumption (piritramide in milligrams) were recorded. Data collection ended with POD 2 in the late evening.

### Pain measurement

The intensity of pain was evaluated by using the NRS (Numeric Rating Scale) Pain Score. The NRS Score is a widely used tool to measure pain intensity by giving numeric values from 0 (no pain) to 10 (maximum pain), allowing general comparability as well as statistical analysis, although some intra- and inter-individual variability might occur [28].

### Statistical analysis

An a priori sample size calculation using BIAS for Windows (Version 11, 2015) was performed. Based on a difference of one grade on the NRS pain score, a significance level of 0.05 and a power of 0.8 (80%), a minimum intention-to-treat group size of 47 patients per group was necessary (effect size d = 0.6; Cohen 1988). We compared differences in pain scores at rest and in motion, knee ROM and opioid consumption between the two groups using the Wilcoxon-Mann–Whitney test. Comparisons of changes in pain scores over time within each group were performed with the Friedman chi-square test, and time to first opioid consumption by using the Log-rank-test (Cox–Mantel and Peto–Pike). Differences between groups were considered significant at P < 0.05.

## Results

Of 139 patients initially designated for the study, 13 patients of the LIA group and 22 patients of the Femoralis group had to be excluded, because of incomplete data records. This was due to the fact that some patients could not be seen, and the relevant data could not be collected on time when there was a high workload for the team on a weekend or late in the afternoon. No patients had to be excluded due to local anaesthetic allergy or due to preoperative opioid intake. Therefore, the data records of 104 patients could be analysed. Besides this high dropout rate, luckily no infections, catheter dislocations, any other relevant problems with the catheters, which would have resulted in exclusion of the patient from the study, could be seen. No infections occurred. We could not detect relevant bleedings in both groups nor was any nerve damage reported. For patient characteristics, see Table 2.

**Table 2:** Patient characteristics

Groups	LIA (*n* = 55)	FEM (*n* = 49)
Female	38 (69.1%)	34 (69.4%)
Male	17 (30.9%)	15 (30.6%)
Age	70.21	68.45
Height	168 cm	166 cm
Weight	85.70 kg	88.61 kg
BMI	30.32	32.01
ASA I	0	2
ASA II	46	34
ASA III	9	13

(patient characteristics presented in % and mean value)

LIA group (local infiltration analgesia)

FEM group (continuous femoral nerve block + sciatic single shot)

We found no significant differences (P > 0.05) in pain scores between the two groups at rest and in motion at all investigation points during the first 48 h. Nor did we find any significant differences (P > 0.05) over time within each group.

**Fig. 1:**
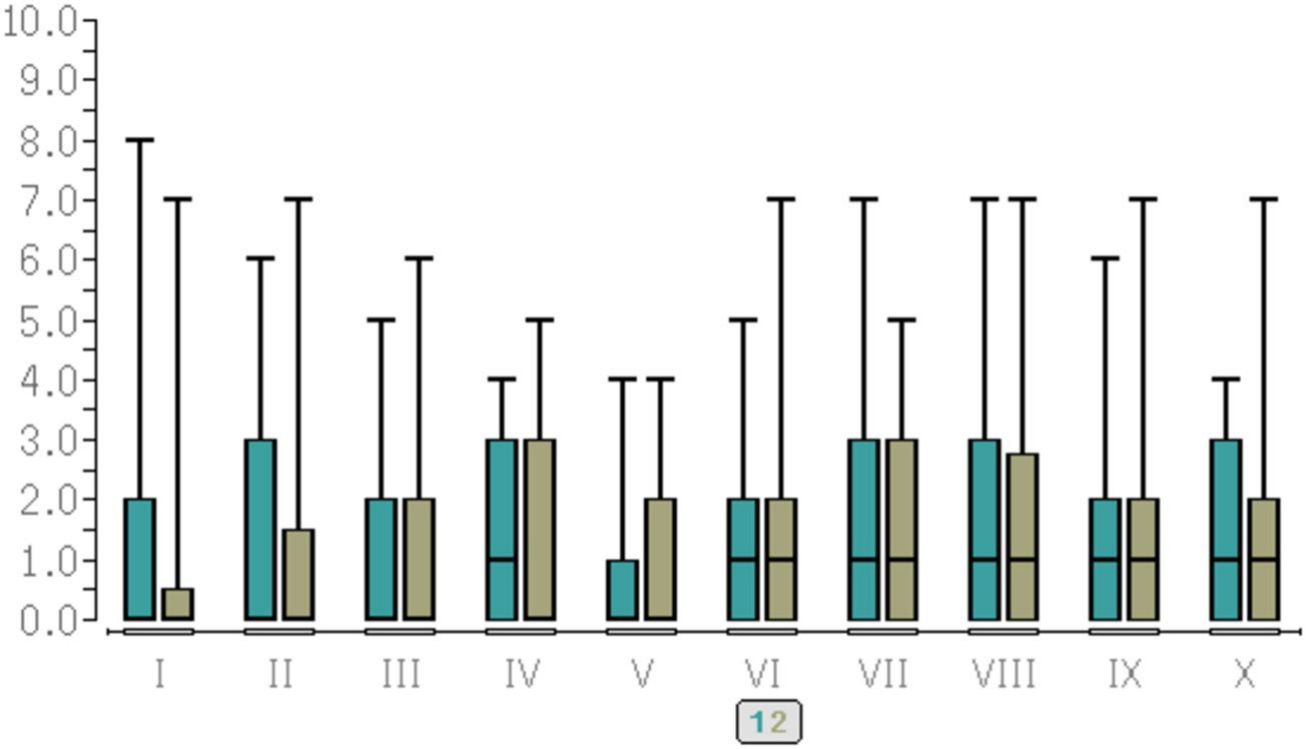
pain scores (NRS) at rest, anterior aspect of the knee

**Fig. 2:**
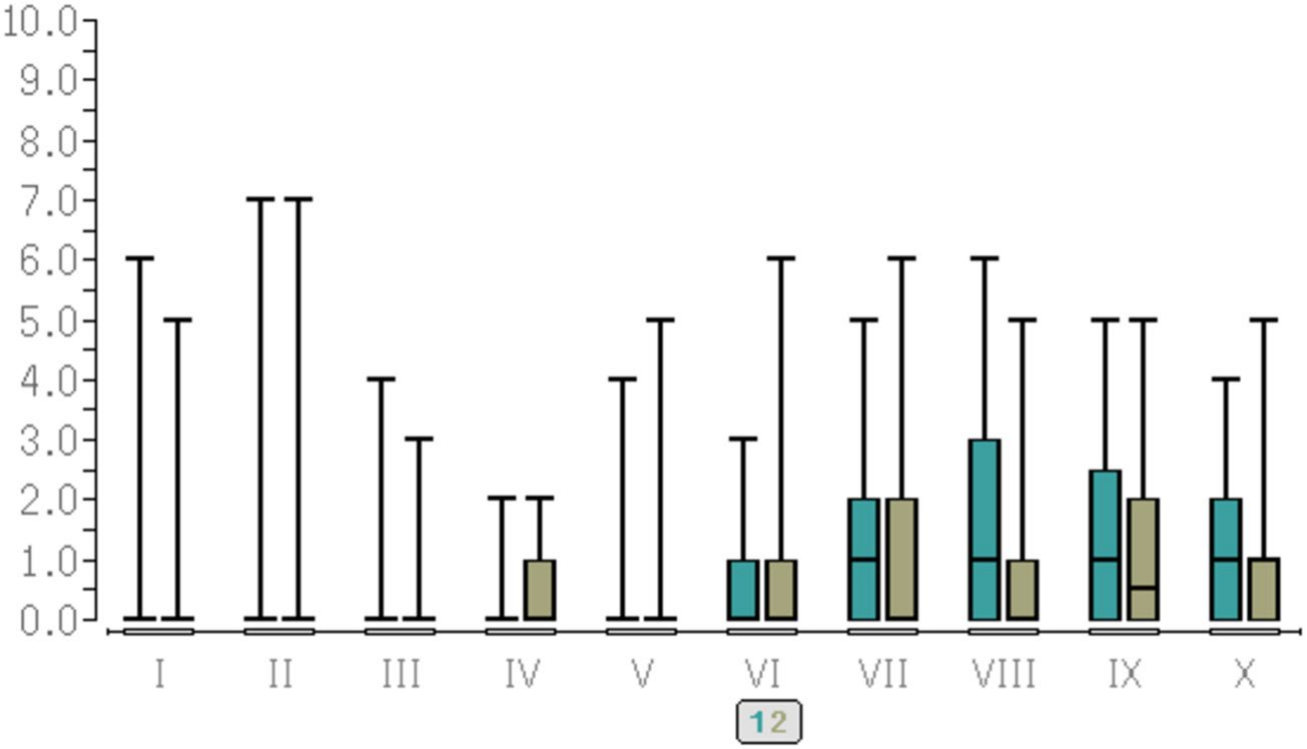
pain scores (NRS) at rest, posterior aspect of the knee. Y-axis = pain scores (NRS). X-axis = time points of evaluation. I = at the end of the operation. II = after one hour in the recovery room. III = after two hours in the recovery room. IV = after three hours in the recovery room. V = at discharge to the ward. VI = at 20:00 on the ward. VII = at 08:00 on POD 1. VIII = at 20:00 on POD 1. IX = at 08:00 on POD 2. X = at 20:00 on POD2. 1 = LIA group, green colour, left bars. 2 = FEM group, brown colour, right bars

For pain scores, see Figs. 1, 2 as well as Table 3.

**Table 3:** Pain scores, median values (Numeric Rating Scale)

Groups	LIA	FEM
	(min./median/max.)	(min./median/max.)
End of operation		
Anterior	0/0/8	0/0/7
Posterior	0/0/6	0/0/5
Recovery room 1		
Anterior	0/0/6	0/0/7
Posterior	0/0/7	0/0/7
Recovery room 2		
Anterior	0/0/5	0/0/6
Posterior	0/0/4	0/0/3
Recovery room 3		
Anterior	0/1/4	0/0/5
Posterior	0/0/2	0/0/2
Discharge to ward		
Anterior	0/0/4	0/0/4
Posterior	0/0/4	0/0/5
On ward at 20:00		
Anterior	0/1/5	0/1/7
Posterior	0/0/3	0/0/6
Maximum pain at rest		
Anterior	0/1/4	0/1/7
Posterior	0/0/2	0/0/6
Maximum pain under movement		
Anterior	0/2/8	0/1/7
Posterior	0/0/4	0/0/6
POD 1 at 08:00		
Anterior	0/1/7	0/1/5
Posterior	0/1/5	0/0/6
POD 1 at 20:00		
Anterior	0/1/7	0/1/7
Posterior	0/1/6	0/1,5/5
Maximum pain at rest		
Anterior	0/1/6	0/1,5/6
Posterior	0/1/6	0/0/4
Maximum pain under movement		
Anterior	0/3/8	0/3/8
Posterior	0/2/8	0/2,5/7
POD 2 at 08:00		
Anterior	0/1/6	0/1/7
Posterior	0/0,5/5	0/1/5
POD 2 at 20:00		
Anterior	0/1/4	0/1/7
Posterior	0/1/4	0/1/5
Maximum pain at rest		
Anterior	0/1/4	0/1/5
Posterior	0/1/4	0/1/5
Maximum pain under movement		
Anterior	0/3/8	0/3/10
Posterior	0/1/6	0/1/7

Pain scores, median values (Numeric Rating Scale, NRS)

*Min.* minimal pain on the NRS

*Median *median pain on the NRS

*Max.* maximum pain on the NRS

For the opioid consumption, there was no significant difference (P > 0.05) within the first 48 h. After the LIA catheter was removed, the LIA group showed higher opioid consumption (piritramide in milligrams) than the FEM group.

**Fig. 3:**
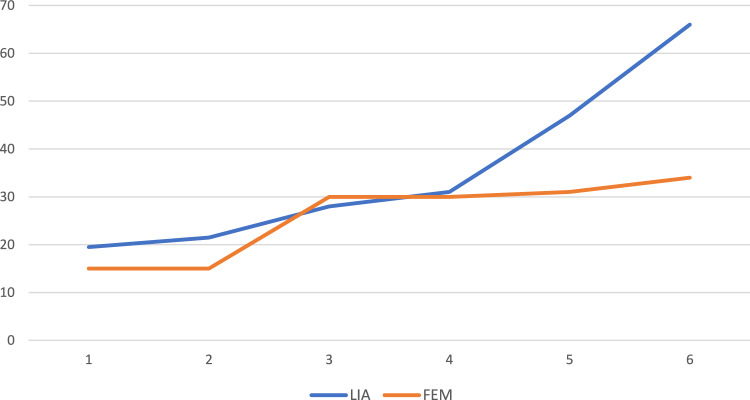
Opioid consumption (piritramide in milligrams). *LIA* LIA Group, local infiltration analgesia. *FEM *Femoralis group. *POD *Post-operative day. X-axis = time points: 1 = discharge to ward. 2 = ward at 20:00. 3 = POD 1 at 08:00. 4 = POD 1 at 20:00. 5 = POD 2 at 08:00. 6 = POD 2 at 20:00. Y-axis: total opioid consumption given in milligrams

**Fig. 4:**
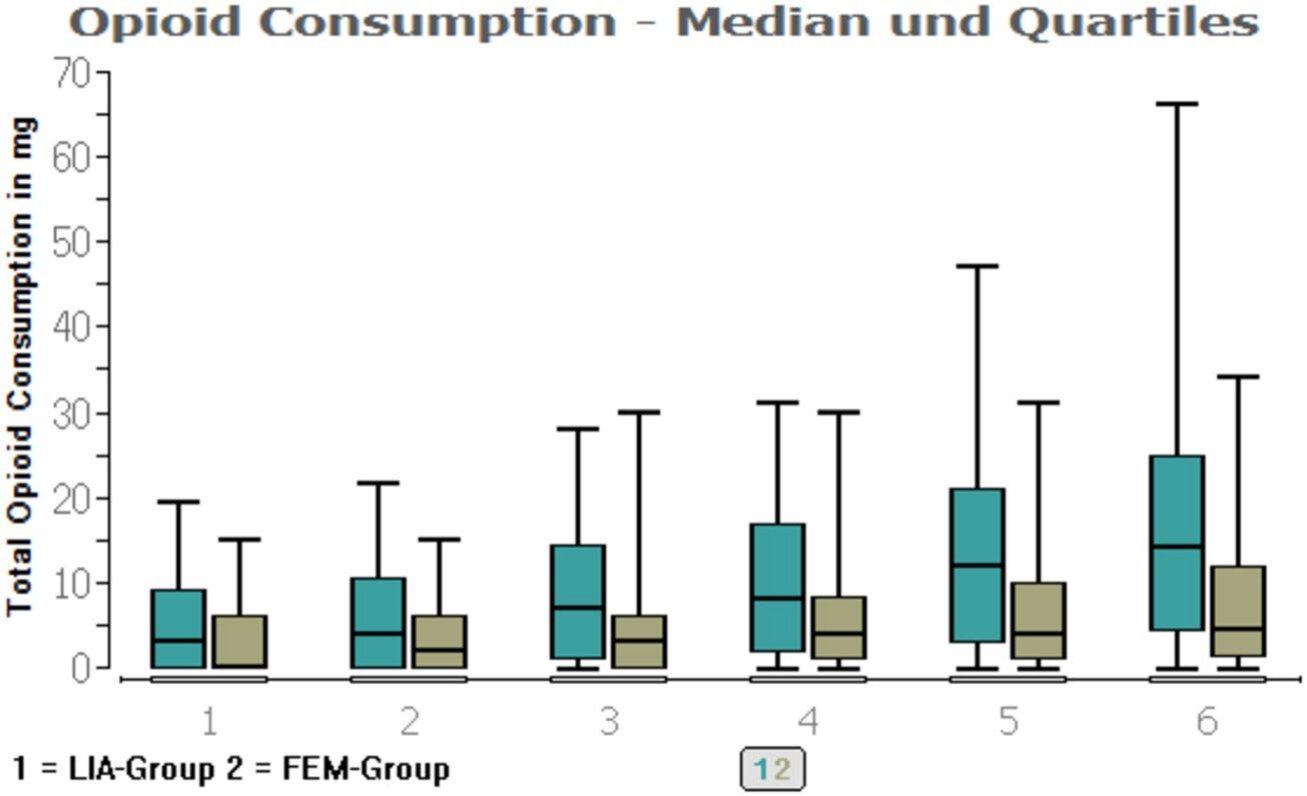
Opioid consumption (piritramide in milligrams). *LIA* LIA group, local infiltration analgesia. *FEM *Femoralis group. *POD* post-operative day. X-axis = time points: 1 = discharge to ward. 2 = ward at 20:00. 3 = POD 1 at 08:00. 4 = POD 1 at 20:00. 5 = POD 2 at 08:00. 6 = POD 2 at 20:00. Y-axis: total opioid consumption given in milligrams

For the opioid consumption (piritramide in mg) see Table 4 and Figs. 3, 4.

**Table 4:** Opioid consumption (piritramide in milligrams)

Groups	LIA	FEM	*P* values
	(min./median/max.)	(min./median/max.)	
End of recovery room	0/3/19.5	0/0/15	*P* = 0.205
Day of operation at 20:00	0/4/21.5	0/2/15	*P* = 0.065
POD 1 at 08:00	0/7/28.0	0/3/30	*P* = 0.082
POD 1 at 20:00	0/8/31.0	0/4/30	*P* = 0.075
POD 2 at 08:00	0/12/47.0	0/4/31	*P* = 0.0093
POD 2 at 20:00	0/14/66.0	0/4.5/34	*P* = 0.0008

Opioid consumption in total, given in milligrams

*POD* postoperative day

*Min.* minimal dose of opioids in milligrams

*Medium *medium dose of opioids in milligrams

*Max.* maximum dose of opioids in milligrams

When analysing the knee ROM, no significant difference (P > 0.05) between the two groups could be found, although a minimal advantage was detectable in the LIA group. On POD 1, the LIA group showed a minimum ROM of 20°, while the FEM group showed a minimum of 0°. ON POD 2, the minimum ROM in the LIA group was 20°, but in the FEM group it was 30°.

**Fig. 5:**
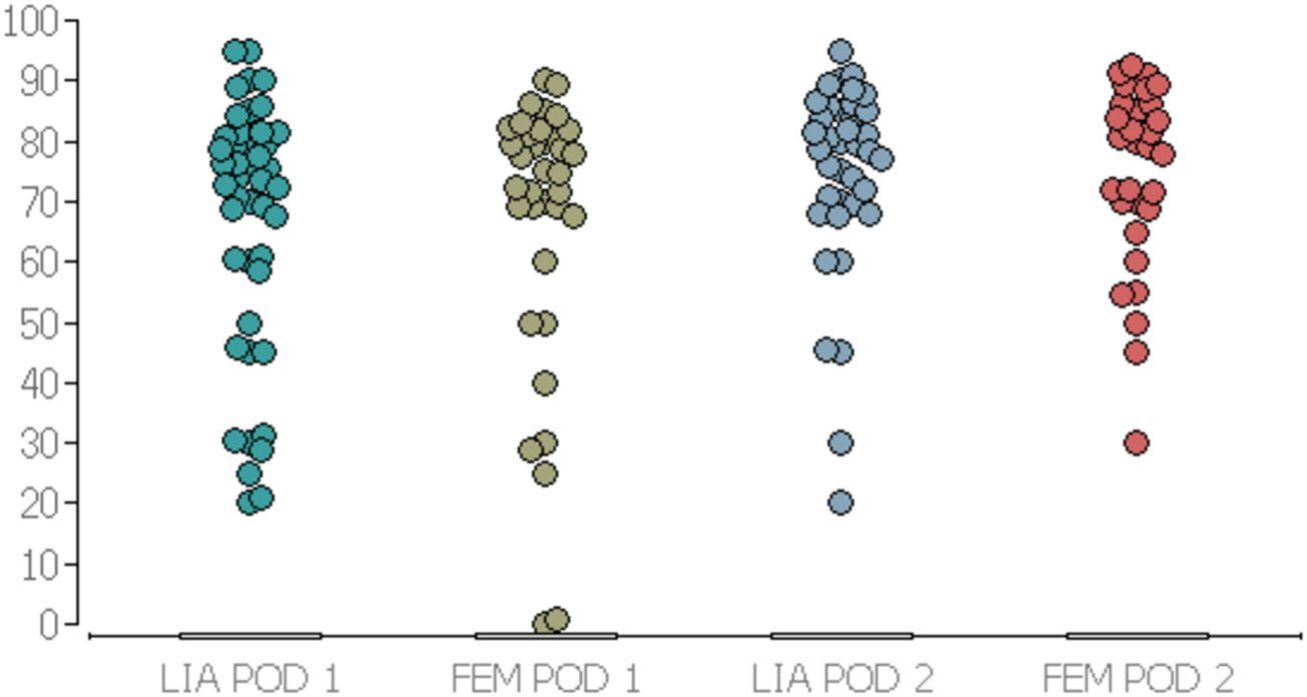
Knee range of motion in degrees. Y-axis = ability of knee movement in degrees. X-axis = LIA POD 1 = LIA group on postoperative Day 1. = FEM POD 1 = Femoralis group on postoperative day 1. = LIA POD 2 = LIA group on postoperative day 2. = FEM POD 2 = Femoralis group on postoperative day 2. (All timepoints: late morning on POD 1 and POD 2)

For the knee ROM, see Table 5 and Fig. 5.

**Table 5:** Knee range of motion in degree

Groups	LIA	FEM	*P* values
	(min./median/max.)	(min./median/max.)	
POD 1	20/75/95	0/75/90	*P* = 0.76
POD 2	20/75/95	30/80/90	*P* = 0.92

*Min.* minimal degree of knee joint flexibility

*Median* average degree of knee joint flexibility

*Max.* Maximum degree of knee joint flexibility

## Discussion

The most important finding of the study was that both techniques showed an equal capacity for pain relief following TKA. The second most important finding of the study was the increase in opioid consumption in the LIA group after removal of the intraarticular catheter on the second day following the operation. This indicates that the catheter worked well; and that there is a demand for pain relief in the form of a need for local anaesthetic after the second postoperative day. A continuous LIA is not recommended according to the PROSPECT recommendations 2020 for TKA [29]. To investigate this ongoing demand for local anaesthetic, a longer observation period seems to be necessary. In this context, the intravenous administration of 8–10 mg of dexamethasone, as well as paracetamol and cox-2-inhibitors, might have contributed to a superior pain relief.

This study was conducted to investigate the potency of LIA compared to the combination of a continuous femoral nerve block (FNB) and a single-shot sciatic nerve block (SNB) for patients undergoing total knee arthroplasty (TKA) under general anaesthesia. Previous studies have compared the LIA with other regional anaesthetic techniques for TKA. These trials proved the effectiveness of the LIA. To our knowledge, no prospective, randomised controlled trial comparing LIA with continuous femoral nerve block + single shot sciatic nerve block under general anaesthesia has been conducted so far.

Lützner et al. [30] compared a combination of continuous FNB + continuous SNB + single shot obturator nerve block to continuous LIA under general or spinal anaesthesia.

Pain scores were analysed for the posterior aspect of the knee separately to evaluate the efficacy of a single-shot sciatic nerve block compared to LIA. Both techniques showed similar pain relief of the posterior aspect of the knee. Patients in the LIA group received ketorolac as a component of the LIA mixture, whereas patients in the Femoralis group did not receive ketorolac. This likely contributed to the better pain relief in the LIA group. Recent studies investigated the efficacy of iPACK [10, 18, 31] and an adductor canal block (ACB) [17] for TKA, showing good pain relief with a reduced risk of motor weakness [32–35] as well as earlier mobilisation [36–39]. The PROSPECT recommendations 2020 for TKA suggest a combination of ACB and LIA [29].

The performance of nerve blocks such as FNB, SNB and iPACK requires more experience, equipment and time than LIA. This suggests that LIA as a method is easier and faster to perform, with a similar effect and lower risk of nerve damage, motor weakness and risk of falls, compared to regional anaesthesia, especially femoral nerve block and proximal adductor canal block.

In contrast, continuous nerve blocks carry the advantage of prolonging the analgesic therapy according to the patient´s needs. In addition, nerve blocks bear no risk of severe intraarticular infections, compared with LIA.

Furthermore, there is a higher risk of overdosing local anaesthetic (LA) with LIA compared to nerve blocks, which may result in higher plasma levels of LA [26, 40, 41]. However, the risk of severe side effects due to high LA levels seems to be low to moderate, especially when adrenaline is used as an adjunct to the LIA mixture [26].

Finally, the potential risk of local anaesthetic-induced chondrotoxicity needs to be addressed as a side effect of LIA when unicompartmental arthroplasty is performed. Differences have been described between several local anaesthetics, which also appear to be both time- and dose-dependent [42, 43]. There is still debate over which technique is optimal for TKA.

We hope our study contributes to this field of knowledge. The limitations of our study are the non-blinded study design, the small sample size, and the relatively short observation time. In retrospect, the study would ideally have been blinded. The study was not blinded to reduce any unnecessary risk of nerve damage or infections in both groups (nerve blocks and LIA could have been performed with NaCl 0,9% instead of local anaesthetic in both groups). Our local ethics committee was not willing to accept this unnecessary risk. Longer observation periods would have allowed identification of the development of chronic pain and success in mobilisation. We also should have focused more on the impact the techniques employed had on early postoperative mobilisation. More details about this aspect are desirable as they are of great clinical importance. As mentioned earlier, the use of ketorolac and its subsequent pain reducing effects in the LIA-group might be a confounding effect.

Furthermore, the abrupt stop of the LIA catheter on the one hand as well as the individual use of the femoral catheter on the other hand could be identified as confounding effects.

## Conclusion

Both techniques had similar pain relief performance at rest and under stress, and there was no significant difference in opioid consumption until the LIA catheter was withdrawn, which prompted higher opioid consumption in the LIA group. Furthermore, both groups showed no significant difference in knee ROM. LIA appears to be a method which is safe and quick to perform. If performed under strict aseptic conditions, the risk of intraarticular infections is low. In our opinion, it is the recommended technique. In addition, the effectiveness of the combination of LIA without catheter + a single shot saphenous nerve block for TKA is a combination which results in no motor weakness and little risk of intraarticular infections. It works well in the institution of our partner clinic as patients are able to ambulate with assistance a few hours postoperatively without any pain in the relevant area. This is consistent with the PROSPECT recommendations in 2020 for TKA, in which the combination of LIA + ACB is the recommended procedure.

## Data Availability

The datasets used and/or analysed during the current study available from the corresponding author on reasonable request.
